# The Effect of Broiler-Targeted Lighting Programs on Productivity, Health, and Welfare

**DOI:** 10.3390/ani16193127

**Published:** 2026-10-06

**Authors:** Parker B. Watson, Emily Jiral, Melinda Grimes, Gregory S. Archer

**Affiliations:** Department of Poultry Science, Texas A&M University, College Station, TX 77845, USA; pbwatson9997@tamu.edu (P.B.W.);

**Keywords:** broiler, dynamic lighting, LED, light spectrum, welfare, corticosterone, tonic immobility, fear, stress

## Abstract

Background: Poultry are highly sensitive to lighting photoperiod, intensity, and color, yet little research has combined these components into a single broiler-targeted lighting program. This study evaluated an integrated dynamic LED lighting program and a further red-light dawn/dusk addition on broiler productivity, health, and welfare. Methods: In Experiment 1, 960 male Cobb 500 broilers were reared for 42 d under a conventional white-LED program (CON) or a dynamic blue-emphasis LED program with declining intensity and a gradually shortening photoperiod (i.e., a lengthening dark period; DYN). In Experiment 2, 960 broilers were reared for 35 d under DYN or DYN with a gradual red-light dawn/dusk transition (RDW). Growth performance, tibia strength and ash, intestinal morphology, blood corticosterone, the heterophil-to-lymphocyte ratio, physical asymmetry, tonic immobility, and inversion were measured, and an illustrative income-over-feed-cost analysis was performed. Results: DYN broilers had higher body weight and lower feed conversion ratio than CON broilers (*p* = 0.016 and *p* = 0.003, respectively), together with a lower heterophil-to-lymphocyte ratio, corticosterone, and composite asymmetry (*p* < 0.05). RDW broilers had greater tibia breaking strengths and lower corticosterone, heterophil-to-lymphocyte ratios, and asymmetry than DYN broilers (*p* < 0.05), with similar growth performance. Illustrative economic analysis suggested a modest income-over-feed-cost advantage for DYN over CON. Conclusions: An integrated, broiler-targeted dynamic lighting program improved growth, intestinal morphology, and stress/fear indicators relative to a conventional program, and adding a red dawn/dusk transition further improved skeletal and stress outcomes without compromising growth, supporting the practical, commercial-scale use of programmable LED technology to improve broiler welfare and productivity.

## Simple Summary

Commercial broiler chickens spend their entire lives under artificial lighting, yet many commercial lighting programs have historically prioritized energy efficiency and operating cost rather than the biology of the birds themselves. This study tested whether a lighting program specifically tailored to broilers, combining light color, brightness, and daily schedule, could improve broiler growth and welfare. In the first experiment, chickens raised under a dynamic blue-spectrum LED program grew faster, converted feed more efficiently, had a healthier gut lining, and showed lower stress and fear compared to birds under a conventional lighting program. A second experiment found that adding a gradual red-light transition at dawn and dusk gave birds stronger leg bones and even lower stress, without slowing growth. Because leg problems are among the most common welfare issues in commercial broiler production, these findings suggest that broiler-targeted lighting programs, built on technology that is already affordable and widely available, could improve both productivity and welfare on commercial farms today.

## 1. Introduction

Poultry are extremely sensitive to even minor components of environmental lighting. Photoperiod, light intensity, and color can all impact physiology and behavior, with even slight deviations leading to detrimental effects on welfare and production [1]. Light-emitting diodes (LEDs) have been reported to provide broiler-appropriate lighting at a lower energy operating cost than conventional incandescent or fluorescent sources [2], making programmable lighting a potentially cost-effective tool for improving poultry production; however, the direct economic comparison of LED technologies was outside the scope of the present study. With innovative lighting technology constantly being developed, research has continued to investigate the effects of manipulating lighting programs on production and welfare.

Several factors must be considered when evaluating a lighting program. Photoperiod, light spectrum, and lighting intensity have all been demonstrated to be key factors for efficient and optimum poultry production. Various studies have concluded that lighting can influence stress responses [3,4], reproductive parameters [5], growth and development [6,7], and fear responses [3,8,9]. Innovation in lighting technology has made it possible to manipulate the environment and positively impact welfare and growth. Building on this literature, the dynamic program evaluated here (DYN) combined a blue-emphasis spectrum, a high initial light intensity that declined with age, and a dark period that lengthened gradually (photoperiod shortening from 23L:1D at placement to 18L:6D by d 20)—a combination selected to more closely approximate the light environment broilers would experience under naturalistic dawn-to-dusk conditions, rather than a fixed industry-standard schedule.

Lewis and Morris [10] concluded that broilers reared under red lighting had inferior performance compared to those reared under blue or green light. Photoperiods have also been shown to influence feed and water intake and improve feed conversion, with a study by Buyse et al. [11] showing intermittent lighting outperforming continuous lighting in these categories. Riaz et al. [12] determined that the proper photoperiod can stimulate growth and performance in commercial broilers. Seo et al. [13] demonstrated that broilers reared under pure blue or bright blue light had significantly larger body weights compared to those reared under pure white light, and also showed improved immune function. Blue and green lighting has been shown to improve welfare by decreasing levels of stress and fear [3,14,15]. Bright red light was shown to increase locomotor activity compared to blue light [16]. One study concluded that birds reared under a dimming period prior to darkness had better tibia symmetry compared to broilers reared under an abrupt light-to-dark transition [17]. When accounting for a color-transitioning lighting program, the rate of transition must also be considered, as poultry are highly sensitive to minor changes in lighting [18].

Most research concerning lighting is focused on how it improves growth and production, rather than the welfare of the birds being produced [19]. Broilers reared under a blue lighting program have been shown to exhibit low fear and stress susceptibility compared to broilers reared under other colors [20]. Archer [2] concluded that broilers reared under a 5000 K color temperature bulb had lower stress expressed by lower asymmetry, corticosterone, and heterophil-to-lymphocyte ratios compared to a 2700 K color temperature bulb. However, there has been some contradiction in results, with Sayin et al. [8] finding that broilers reared under green-blue combined lighting had higher fear levels compared to those under green, blue, or blue-green combined lighting, suggesting that the presentation of lighting colors may have an impact on poultry welfare.

While there is an abundance of research on lighting effects in broilers, there has been little work creating an all-encompassing program specifically designed for broilers. Therefore, the objective of this study was to assess the impact of a specifically tailored lighting program on broiler stress, fear, health, and growth. The first experiment compared a traditional lighting program to a dynamic program designed with spectrum, intensity, and photoperiod simultaneously considered. A second experiment was conducted with the addition of a dawn/dusk period to the experimental program to determine if it would have additional effects. It was hypothesized that rearing broilers under an integrated dynamic lighting program combining a blue-emphasis spectrum, declining intensity, and a gradually shortening photoperiod (intended to approximate naturalistic dawn-to-dusk lighting more closely than a fixed industry-standard schedule) would improve growth and health parameters while diminishing levels of fear and stress; because these components varied together, this hypothesis was tested at the level of the integrated program rather than for individual lighting components.

## 2. Materials and Methods

### 2.1. Animals and Husbandry

All methods adhered to the standards set by the Texas A&M Institutional Animal Care and Use Committee (AUP# 2021-0313) as well as the Guide for the Care and Use of Agricultural Animals in Research and Teaching [21]. The broilers were kept at the Texas A&M University Poultry Science Teaching, Research, and Extension Center, where all diets were prepared locally.

Experiment 1. A total of 960 male Cobb 500 chicks (Cobb-Vantress, Siloam Springs, AR, USA) were sourced from a commercial hatchery and raised over a period of 42 days. The study compared two lighting treatments: (1) a standard white-LED lighting setup (CON; ONCE by Signify, Plymouth, MN, USA; Table 1) and (2) a dynamic LED lighting system designed specifically for broilers (DYN; Once dynamic LEDs, ONCE by Signify, Plymouth, MN, USA; Table 2). The experiment was structured as two separate trials conducted at different times, each involving 480 chicks. In each trial, 24 floor pens (measuring 1.22 m × 1.83 m, with 20 chicks per pen) were assigned to the two treatments, resulting in 12 pens per treatment per trial and a total of 24 pens per treatment (480 birds per treatment) across both trials. On the day of hatching, chicks were randomly selected, weighed to ensure uniform starting weights across pens, and placed at a density of 20 chicks per pen. The pen served as the experimental unit.

Throughout both experiments, birds had unrestricted access to both feed and water. The diets, based on corn and soybean meal, were designed to meet or surpass the nutrient guidelines for Cobb 500 broilers and were prepared at the Texas A&M University feed mill. These diets were administered in three stages: the starter phase (days 0–13, crumble form), the grower phase (days 13–21, pellet form), and the finisher phase (days 21–42 for Experiment 1 and days 21–35 for Experiment 2, pellet form). Table 3 details the ingredients and calculated nutrient content of each diet. Each pen was equipped with a single hanging tube feeder for feed distribution, while water was provided via a nipple drinker line featuring three nipple drinkers per pen. The pens were lined with fresh pine-shavings litter, and the temperature and ventilation were managed according to the Cobb 500 management guide.

**Figure 1:**
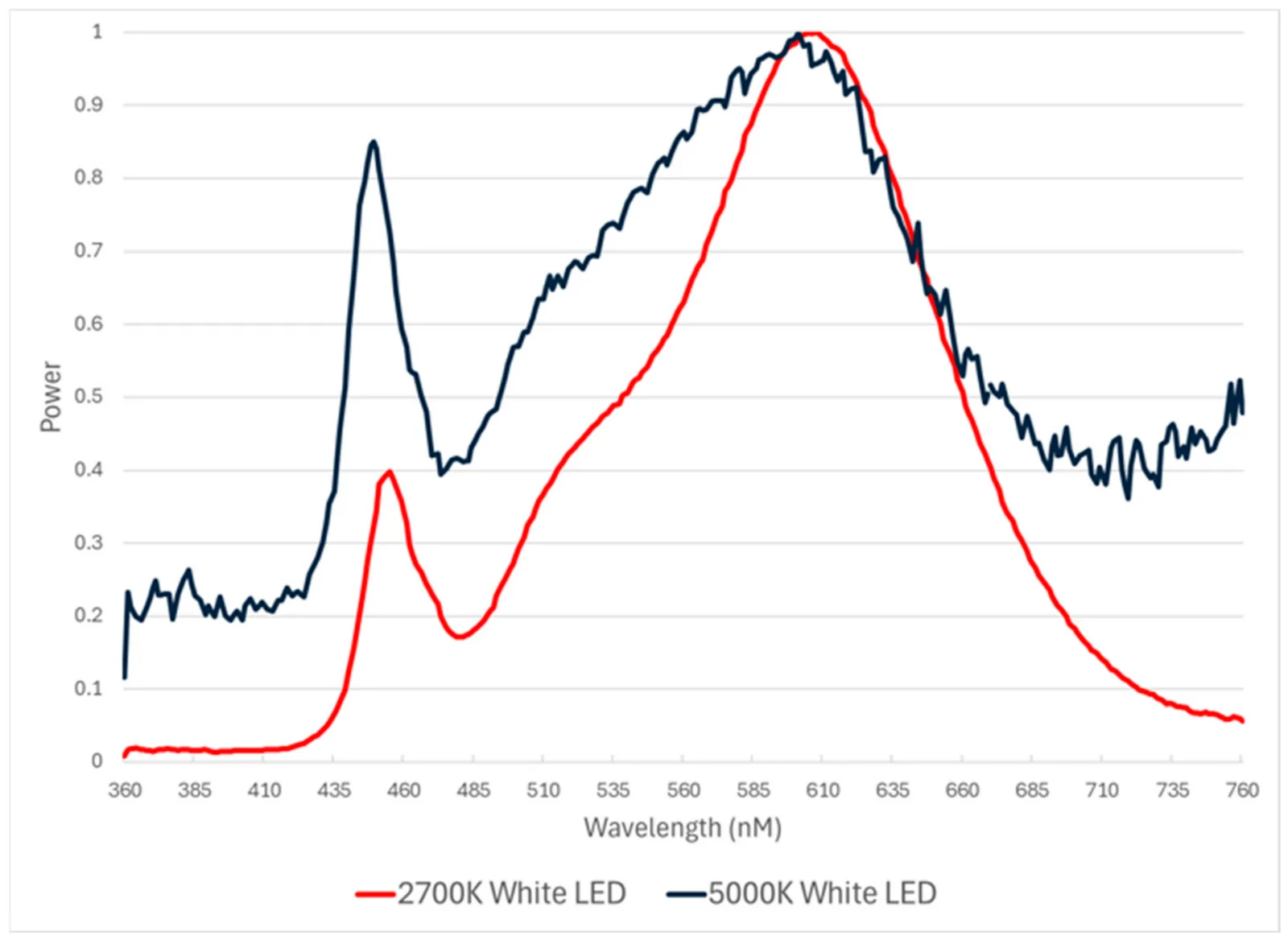
Spectral Output of CON Lighting Treatment; 2700 K d0-5 and 5000 K d6-end of trial.

**Figure 2:**
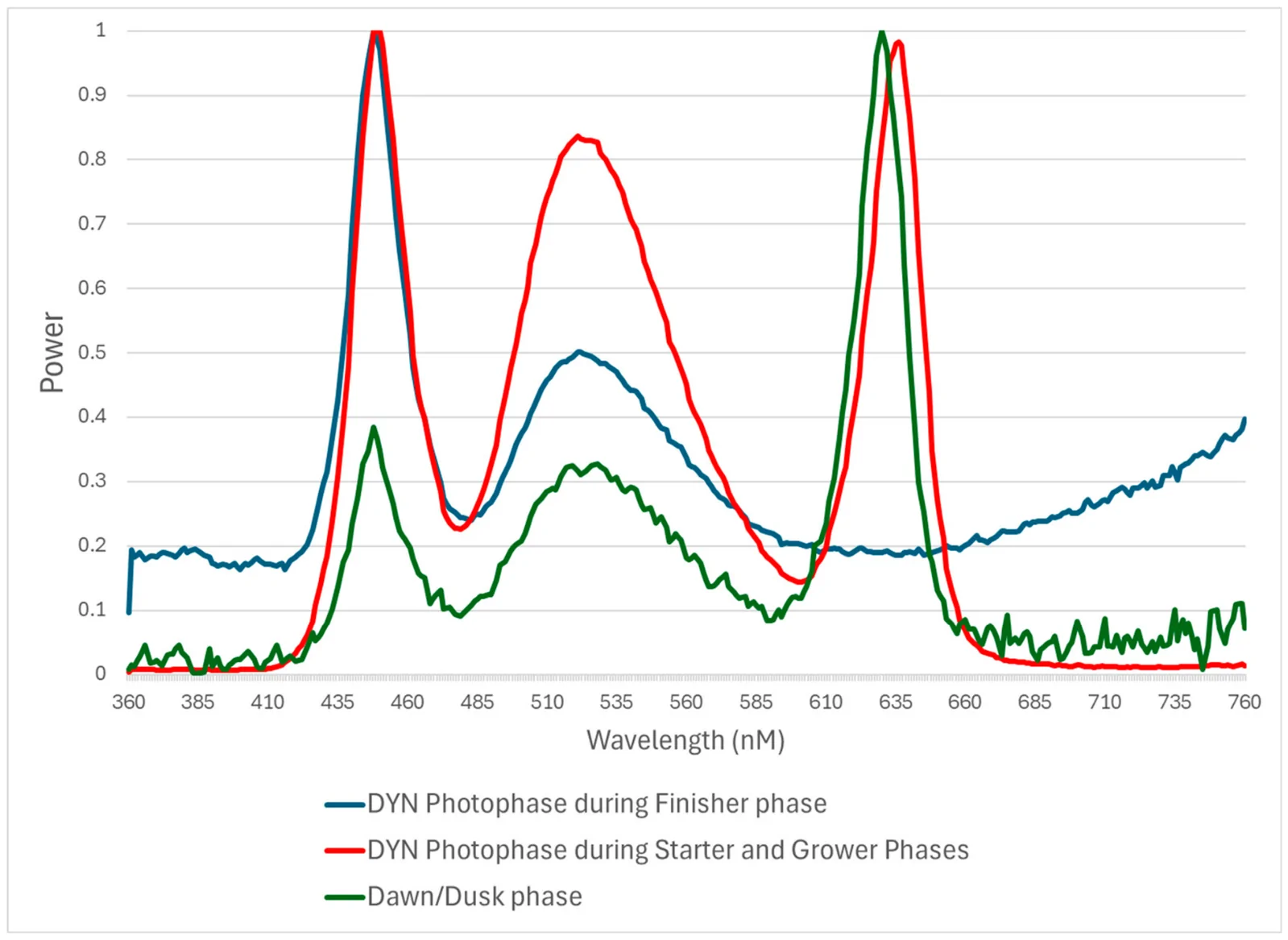
Spectral Output of DYN Lighting Treatment, Starter/Grower Phases (d0-19), Finisher.

The CON treatment utilized white LED bulbs (ONCE by Signify, Plymouth, MN, USA) installed in fixtures, while the DYN treatment employed dynamic LED bulbs (ONCE by Signify, Plymouth, MN, USA), positioned directly above the pens at a height of 3 m from the floor. The CON lights were managed with a single dimmer and timer, whereas the DYN lights were operated through the Interact Agriculture (Signify Netherlands B.V., Eindhoven, The Netherlands) application. During the initial 24 h post-placement, the CON group was exposed to a light intensity of 25 lux, using bulbs with a color temperature of 2700 K, and maintained a photoperiod of 24L:0D, as measured at broiler height with a light meter (UPRtek Mk350, UPRtek, Zhunan, Taiwan). At six days old, the lighting was adjusted to 5000 K. Details and schedules of changes in photoperiod, light intensity (in lux), and color temperature (in Kelvin) for the conventional lighting program are outlined in Table 1 and Figure 1 and Figure 2. The DYN program was configured to emit a blue color, with an average wavelength of 450 nm. For the first 24 h after placement (day 0), the DYN group was kept under continuous light (24L:0D) at 75 lux, measured from broiler head height using a light meter (UPRtek Mk350, UPRtek, Zhunan, Taiwan). A summary of the photoperiod schedule and lux for the DYN treatment is provided in Table 2. The spectral output of the DYN lighting program is depicted in Figure 2. All lighting treatments were separated by barriers to prevent light interference. The CON program (Table 1) was selected to reflect standard U.S. commercial broiler practices, where intensity is reduced to about 5 lux (0.5 foot-candles) after brooding to minimize activity and prevent injurious pecking; 5 lux is the minimum allowed by the National Chicken Council broiler welfare guidelines [22] and is commonly used by U.S. integrators, although it falls below the ≥20 lux minimum specified in Council Directive 2007/43/EC and several retailer welfare standards. Since the CON and DYN treatments varied in intensity, spectrum, and photoperiod, intensity cannot be ruled out as a factor contributing to the observed treatment differences. However, intensities ranging from 1–40 lux have been reported to have no impact on broiler body weight, feed intake, feed conversion, or mortality [23], and the DYN program itself decreased to 10 lux from day 20 onward (Table 2), so the most significant intensity difference between programs (5 vs. 19–75 lux) was limited to days 0–19 and is unlikely to solely explain the growth and welfare differences observed at day 42. As the CON and DYN treatments differed in fixture/control system, spectrum, light intensity, color temperature, and photoperiod schedule (Table 1 and Table 2), Experiment 1 assesses the combined lighting program as a whole; the aim was not to determine which specific component (e.g., spectrum vs. intensity vs. photoperiod) was responsible for the observed treatment differences.

Experiment 2. A total of 960 male Cobb 500 broilers were sourced from a commercial hatchery on the day they hatched and were raised for a period of 35 days. This study evaluated the dynamic lighting program (DYN, as used in Experiment 1) against the same program enhanced with a gradual red-light transition at dawn and dusk at the beginning and end of each lighting period (RDW). Each treatment involved 12 pens, each measuring 1.22 m × 1.83 m, housing 20 birds per pen. The trial was conducted twice over time. Once a weight range was determined, 20 birds were randomly chosen and weighed to populate each of the 24 pens. Throughout the trial, the birds had unlimited access to feed. All feed provided was manufactured by the Texas A&M feed mill. A standard starter diet was given to the flock from day 0 to day 13. On the 13th day, any leftover feed in the feeders was weighed, and a grower diet was then introduced and fed from day 13 to day 21. On day 21, the remaining feed was again weighed, and a standard finisher feed diet was weighed and distributed to all feeders until the trial concluded on day 35, at which point any leftover feed was weighed for the final time. Each pen was equipped with watering lines, providing 3 nipple drinkers per pen.

The DYN and RDW treatments were equipped with dynamic LED light bulbs (ONCE by Signify, Plymouth, MN, USA) positioned 3 m above the floor directly over the pens, managed through the Interact Agriculture (Signify Netherlands B.V.) application. The DYN program mirrored the one used in Experiment 1, but it concluded at day 35 instead of day 42. Table 2 provides a descriptive overview of the DYN program. Figure 2 illustrates the spectral output of the DYN lighting program. The RDW program was similar to DYN, except that at the start of each light period, the spectral output gradually shifted over 15 min to reach a peak red dawn color, then transitioned over another 15 min to the standard DYN spectrum (Figure 2); both treatments adhered to the same intensity and photoperiod schedule as DYN (Table 2). Each light period concluded with a 15-min transition to the peak red dusk color, followed by a further 15-min transition to off. These dawn and dusk transitions were incorporated within the scheduled photoperiod (Table 2) rather than extending it: the 30-min dawn sequence commenced at the scheduled lights-on time, and the 30-min dusk sequence concluded at the scheduled lights-off time, ensuring that the total daily hours of light and darkness remained the same for both DYN and RDW.

### 2.2. Growth Performance and Feed Conversion

In Experiment 1, birds were weighed on days 0 and 42, while in Experiment 2, they were weighed on day 35 to assess the average daily gain. Throughout the trial, feed was measured at distribution and again during feed phase transitions to compute feed intake and the feed conversion ratio. Feed intake was determined by subtracting the leftover feed at the conclusion of the starter, grower, and finisher phases from the total feed provided. The average daily feed intake was calculated by dividing the total feed consumed by the number of birds and the trial days, expressed as grams per bird per day. The feed conversion ratio was obtained by dividing the total feed consumed by the total live-weight gain of the pen, adjusted for mortality. Mortality data was collected daily and weighed to adjust the feed conversion for mortality losses.

### 2.3. Tibia Breaking Strength and Ash Content

In Experiment 1 on day 42 and Experiment 2 on day 35, both the left and right tibiae were randomly collected from 24 birds per treatment group (*n* = 48 birds per treatment, totaling 96 tibiae per experiment, with 48 per treatment, as each bird provided both left and right tibiae). Following euthanasia through cervical dislocation, the tibiae were extracted and stored in labeled sample bags for processing the next day. Initially, the breaking strength of the fresh, intact tibiae was assessed using a 3-point bending test with a TA XT Plus100 Texture Analyzer (Texture Technologies Corporation, Scarsdale, NY, USA). Subsequently, the same bones underwent preparation for tibia ash analysis by removing the fibula, muscle, and connective tissue; drying at 100 °C for 12 h; defatting with diethyl ether for 8 h; air drying; another 12-h drying at 100 °C; and finally ashing in ceramic crucibles at 600 °C for 24 h.

### 2.4. Intestinal Morphology

On day 42 of Experiment 1 and day 35 of Experiment 2, a 2-cm section from the mid-ileum, located between Meckel’s diverticulum and the ileocecal junction, was obtained from one bird per pen (*n* = 12 pens per treatment per experiment). These segments were carefully rinsed with 0.9% saline to eliminate digesta, then fixed in 10% neutral buffered formalin for 24 h. Following fixation, they were dehydrated using a graded ethanol series, cleared, and embedded in paraffin. Transverse sections, each 5 µm thick, were sliced using a microtome, placed on glass slides, and stained with hematoxylin and eosin. The sections were visualized with an imaging system (Nikon Eclipse Ci-LNikon Corporation, Tokyo, Japan) and analyzed using image-analysis software (NIS-Elements, version X.X; Nikon Corporation, Tokyo, Japan). Measurements of villus height were taken from the tip of the villus to the villus–crypt junction, while crypt depth was measured from the villus–crypt junction to the crypt base. For each bird, 10 well-oriented, intact villus–crypt units were measured, ensuring that villi were only included if the lamina propria was intact and a complete crypt was visible. All measurements were conducted by a single observer who was blinded to the treatment. The ratio of villus height to crypt depth was calculated for each villus–crypt unit, averaged per bird, and the resulting bird value was used as the pen observation for analysis.

### 2.5. Blood Measurements

On day 42 of Experiment 1 and day 35 of Experiment 2, a bird was randomly chosen from each pen to assess the heterophil to lymphocyte ratio (HL), corticosterone (CORT) levels, and total white blood cell (WBC) count. Blood samples, ranging from 4 to 5 mL, were drawn from the brachial wing vein of each broiler. Sampling occurred between 08:00 and 11:00 h, which is two hours after the lights were scheduled to turn on. Treatments were sampled in an alternating manner to ensure all were assessed at the same point in the light schedule and within the same time frame. Each bird was individually captured within the pen and bled within one minute to reduce the impact of handling on plasma corticosterone levels. A small drop of blood was immediately smeared on a glass slide for the HL ratio analysis. The remaining blood was divided for different tests; half was placed into a plasma separation gel and lithium heparin vacutainer (BD, Franklin Lakes, NJ, USA), then stored temporarily in an ice bath. The other half was injected into a tube with ethylenediaminetetraacetic acid (EDTA) to prevent coagulation and sent to the Texas A&M Veterinary Medical Diagnostic Laboratory for total WBC count determination (×10^3^ cells/µL). After all samples were collected, vacutainers for corticosterone measurement were centrifuged at 4000 RPM for 15 min to separate plasma from blood cells. The separated plasma was then transferred into a 2-mL microcentrifuge tube and stored at −19 °C until further analysis. Blood smear slides for the HL ratio were stained using a hematology staining kit (Cat# 25034, Polysciences, Inc., Warrington, PA, USA).

Plasma corticosterone levels in each sample were measured using ELISA kits available from Enzo Life Sciences (ADI-901-097, Farmingdale, NY, USA). The coefficient of variation for both inter-assay and intra-assay was below 5%. To calculate the heterophil-to-lymphocyte ratios for each sample, a thin layer of blood cells was spread on a glass slide, stained, and examined under a microscope (Omax DCE-2, Kent, WA, USA) at 40× magnification with an oil immersion lens. A keystroke counter (SEOH B4001-5LC, Navasota, TX, USA) was employed to tally the heterophils and lymphocytes in a non-overlapping area of the blood smear. Counting continued until 100 cells were recorded. Increased plasma corticosterone [24] and heterophil-to-lymphocyte ratios [25] may suggest greater stress susceptibility in poultry.

### 2.6. Physical Asymmetry

In Experiment 1 on day 42 and Experiment 2 on day 35, five birds from each pen were randomly chosen to assess three bilateral foot traits. Measurements for the middle toe length, metatarsal length, and metatarsal width were taken on both the right and left legs using a Craftsman IP54 Digital Caliper (Sears Holdings, Hoffman Estates, IL, USA). To determine the composite asymmetry score, the absolute differences between the left and right sides for each trait were summed and then divided by the number of traits. The formula used in this study is (|L-R|MTL + |L-R|ML + |L-R|MW)/3, resulting in the composite asymmetry score.

### 2.7. Tonic Immobility

Tonic Immobility (TI) data was gathered on the last day of each experiment. In each pen, five birds were randomly chosen for Tonic Immobility assessment. To ensure accurate measurements, the birds were handled with utmost care. The birds were gently turned over so their abdomen faced upwards and placed in a U-shaped cradle cushioned with cloth. After positioning them on their backs, the researcher’s hand was kept on their breast for about 25 s to trigger tonic immobility. For an attempt to be considered valid, the bird needed to stay immobile for a minimum of 10 s. If the bird flipped over within the initial 10 s, it was noted as an attempt, and the bird was repositioned for another try. If three attempts were unsuccessful, a time of 0 s was recorded. If the bird remained immobile beyond the initial 10 s, the first head movement was observed and documented. Subsequently, the time taken for the bird to right itself was monitored, and this final time was recorded. A longer duration to correct position indicates a higher level of fear in the broilers being evaluated. All TI testing was performed by a single individual.

### 2.8. Inversion

Inversion, akin to tonic immobility, serves as another fear assessment gathered from birds at the study’s end. Five birds from each pen were randomly chosen and held upside down by their legs until they stopped flapping or for a maximum of 30 s. Each bird’s inversion was captured on video for later analysis of intensity. Trained observers reviewed the recordings, noting the number of wing flaps and the duration of flapping. By dividing the flap count by the flapping duration in seconds, we calculated the intensity of wing flapping. A higher intensity indicates a stronger fear response. A single individual conducted all the inversion tests.

### 2.9. Statistical Analysis

All data were analyzed using Minitab (version 21; Minitab LLC, State College, PA, USA). Pen was the experimental unit for all analyses; for measurements taken on individual birds (tibia, blood, intestinal morphology, asymmetry, tonic immobility, and inversion), bird values were averaged within pen before analysis. Each experiment consisted of two temporally separated trials. For each response variable, the full model initially fitted using the General Linear Model (GLM) procedure was Y_ijk_ = μ + T_i_ + R_j_ + (T × R)_ij_ + e_ijk_, where T_i_ is the fixed effect of lighting treatment (i = 1, 2), R_j_ is the effect of trial (replicate in time; j = 1, 2), (T × R)_ij_ is the treatment × trial interaction, and e_ijk_ is the residual error. Because the treatment × trial interaction was not significant (*p* > 0.05) for any variable, data from the two trials were pooled and the final analysis was a one-way ANOVA with lighting treatment as the sole fixed effect (*n* = 24 pens per treatment). Pen mortality percentages were analyzed with the same model, and treatment differences in pooled mortality counts were additionally examined by chi-square test. Differences were considered statistically significant at *p* < 0.05, and 0.05 ≤ *p* < 0.10 was considered a tendency.

### 2.10. Production Efficiency and Illustrative Economic Analysis

The European Production Efficiency Factor (EPEF) was calculated for each treatment as EPEF = [Livability (%) × Live Body Weight (kg)]/[Age (d) × Feed Conversion Ratio] × 100, using pen mortality data collected throughout each trial (mortality was corrected for in feed conversion ratio as described above) and the d 42 (Experiment 1) or d 35 (Experiment 2) body weight and feed conversion ratio reported in Table 4 and Table 6. An illustrative income-over-feed-cost (IOFC) partial-budget analysis was also performed to help contextualize the production differences observed. Because treatment-specific feed and live-bird prices were not recorded in this study, IOFC was calculated using representative, non-treatment-specific U.S. broiler-industry benchmark prices for complete feed ($0.38/kg, representative of recent corn–soybean meal-based diet costs) and live body weight ($1.10/kg, informed by USDA 2026 national broiler price forecasts [26]), and is intended only to illustrate the direction and approximate magnitude of the economic implications of the production differences observed rather than to represent a validated economic evaluation of these lighting programs. Total feed consumed per bird marketed was estimated as feed conversion ratio × live-weight gain (final body weight minus an assumed day-old chick weight of 0.042 kg); revenue per bird marketed was estimated as final body weight × live-bird price; and IOFC was calculated as revenue minus feed cost. IOFC per bird placed was additionally estimated by multiplying IOFC per bird marketed by treatment livability to reflect the economic consequence of mortality.

## 3. Results and Discussion

### 3.1. Experiment 1—Growth Performance

Production parameters are presented in Table 4. Broilers reared under the DYN program had higher d-42 body weight (2.99 vs. 2.85 kg; *p* = 0.016), higher average daily gain (*p* = 0.016), and lower feed conversion ratio (1.45 vs. 1.53; *p* = 0.003) compared to CON broilers. Average daily feed intake tended to be greater in CON broilers (101.4 vs. 96.5 g/bird/d; *p* = 0.07). These findings are consistent with previous research demonstrating that optimized photoperiods can stimulate growth and performance in commercial broilers [11,12]. Because CON and DYN differed simultaneously in spectrum, intensity, and photoperiod, the specific contribution of the DYN spectrum’s blue emphasis cannot be isolated from the other program components; it should be noted that the CON white LEDs also emit within the blue region, so any spectral effect would reflect a difference in blue-light emphasis and intensity distribution rather than presence versus absence of blue wavelengths. Blue wavelengths have previously been associated with enhanced body weight and immune function relative to white light in other studies [13], consistent with, though not proof of, a contribution of spectrum to the improvements observed here. The reduction in average daily feed intake observed in DYN broilers, despite superior body weight gain, suggests improved feed utilization efficiency; the economic implications of this improvement are explored in the illustrative partial-budget analysis presented below.

European Production Efficiency Factor (EPEF), which incorporates livability together with body weight, age, and feed conversion ratio, was 437.8 for DYN broilers compared with 411.2 for CON broilers (Table 4). The higher EPEF for DYN broilers indicates that their improved body weight and feed conversion ratio more than offset their numerically higher mortality relative to CON broilers.

Mortality merits separate consideration in a welfare context. Over the 42-d period, mortality was 7.29% (35 of 480 birds placed; 1.46 ± 0.30 birds per pen) for CON and 10.83% (52 of 480; 2.17 ± 0.37 birds per pen) for DYN broilers. The difference between treatments was not statistically significant when analyzed at the pen level (*p* = 0.14; Table 4), although a chi-square test on the pooled counts approached significance (*p* = 0.056). Mortality was higher in the second trial than in the first for both treatments (CON, 8.3 vs. 6.3%; DYN, 15.0 vs. 6.7%; trial *p* = 0.03; treatment × trial *p* = 0.17). Mortality in both treatments occurred predominantly during the first 7 d/after d 28 and was attributed on necropsy to yolk-sac infection, sudden death syndrome, ascites, and leg-related issues; no treatment-specific cause of death was identified. Although not statistically significant, the 3.5-percentage-point numerical difference in livability (89.17 vs. 92.71%) is of practical relevance and should be examined in larger commercial-scale evaluations of the DYN program. Because mortality was recorded daily and dead birds were weighed, feed conversion ratio, EPEF, and IOFC per bird placed all account for these losses.

### 3.2. Experiment 1—Health Parameters

Health measurements are also presented in Table 4. DYN broilers had a lower white blood cell count (*p* = 0.05) and higher villus height-to-crypt depth ratio (*p* = 0.049) compared to CON broilers. No differences were observed in bone ash content or tibia breaking strength (*p* > 0.05). The higher villus-to-crypt ratio in DYN broilers is consistent with improved intestinal morphology, which may be one of several factors contributing to the improved feed conversion observed, although absorptive capacity itself was not directly measured [27].

### 3.3. Experiment 1—Stress and Fear

Stress and fear data are presented in Table 5. DYN broilers exhibited lower stress susceptibility as expressed by the heterophil-to-lymphocyte ratio (0.91 vs. 1.90; *p* = 0.006), composite asymmetry (1.43 vs. 1.76 mm; *p* = 0.007), and plasma corticosterone (1129.8 vs. 2140.3 pg/mL; *p* = 0.01). Elevated corticosterone and heterophil-to-lymphocyte ratios are well-established indicators of chronic stress susceptibility in poultry [24,25], and the magnitude of difference observed is consistent with reduced physiological stress. These findings align with Archer [2], who reported lower corticosterone and heterophil-to-lymphocyte ratios in broilers under cooler color temperature lighting, and with Hesham et al. [20], who observed reduced stress behaviors in broilers under blue lighting programs.

DYN broilers demonstrated shorter tonic immobility latency to right (205.3 vs. 272.2 s; *p* = 0.011), consistent with reduced fear susceptibility [28]. However, DYN broilers exhibited longer inversion duration and a greater number of wing flaps, which may reflect increased activity under blue light conditions rather than heightened fear, when considered alongside the reductions in corticosterone and heterophil-to-lymphocyte ratio. This apparent discrepancy may be attributable to the differential sensitivity of tonic immobility and inversion as measures of fear, as the two tests do not always yield concordant results [29].

### 3.4. Experiment 2—Growth Performance and Health

Production and health parameters from Experiment 2 are presented in Table 6. Broilers reared under the RDW program had greater feed intake (105.94 vs. 102.57 g/d; *p* = 0.018) compared to DYN broilers; all other production parameters including body weight, average daily gain, and feed conversion ratio were similar between treatments (*p* > 0.05). The marginally greater feed intake in RDW broilers without a corresponding improvement in body weight could reflect increased activity during transition periods, consistent with Prayitno et al. [16], who reported elevated feeding and locomotor activity under red illumination; however, locomotor activity was not directly measured in the present study, so this explanation should be regarded as a hypothesis rather than a demonstrated mechanism. RDW broilers had greater tibia breaking strength (38,951 vs. 30,650 g; *p* < 0.001) than DYN broilers, with no difference in bone ash content (*p* > 0.05). This dissociation between breaking strength and mineral content may suggest that the observed difference in skeletal integrity may reflect differences in bone geometry or collagen matrix quality rather than mineralization per se; however, neither bone geometry nor collagen composition was directly measured, and this interpretation should be treated as a hypothesis for future investigation. We acknowledge that a difference of this magnitude (approximately 27%) in response to a 30-min spectral transition at each end of the photoperiod is larger than would be anticipated and that it was observed in a single experiment; the result should therefore be interpreted with caution and requires confirmation in independent trials, ideally with measurement of bone geometry, before any causal inference is drawn. The red dawn/dusk periods may have promoted bone remodeling through increased locomotor activity and mechanical loading, although activity was not directly measured [30]. Van der Pol et al. [17] similarly observed improved tibia symmetry in broilers exposed to gradual light-to-dark transitions, lending further support to the notion that naturalistic lighting transitions benefit skeletal development.

A trend toward a lower villus-to-crypt ratio was observed in RDW broilers compared to DYN broilers (*p* = 0.058). The mechanism by which red dawn/dusk periods may influence intestinal morphology is not immediately apparent and warrants further investigation.

EPEF was 440.5 for DYN broilers (93.13% livability) compared with 429.3 for RDW broilers (93.75% livability; Table 6). Despite comparable body weight and slightly higher livability, the numerically higher feed conversion ratio of RDW broilers resulted in a lower EPEF than DYN broilers. Mortality did not differ between treatments (6.88 vs. 6.25% for DYN and RDW, respectively; 33 and 30 of 480 birds placed; *p* = 0.77).

### 3.5. Experiment 2—Stress and Fear

Stress and fear data from Experiment 2 are presented in Table 7. Broilers reared under the RDW program exhibited lower asymmetry (1.50 vs. 1.77 mm; *p* = 0.018), heterophil-to-lymphocyte ratios (0.53 vs. 0.81; *p* < 0.001), and plasma corticosterone (11,648.4 vs. 25,062.6 pg/mL; *p* = 0.016) compared to DYN broilers. The gradual transition from red to blue light at the onset and offset of each photoperiod may more accurately replicate the spectral shift observed during natural sunrise and sunset, potentially attenuating the acute stress response associated with abrupt light onset [18]. It should be noted that absolute plasma corticosterone concentrations in Experiment 2 were considerably higher than those in Experiment 1 (Table 5 and Table 7), even though the DYN program was common to both. The two experiments were conducted at different times of year with different bird cohorts; birds were sampled at different ages (d 35 vs. d 42).

RDW broilers demonstrated significantly shorter tonic immobility time to first head movement (131.1 vs. 185.0 s; *p* = 0.017) and latency to right (201.3 vs. 255.1 s; *p* = 0.031) compared to DYN broilers, consistent with lower fear susceptibility. No differences were detected between treatments during the inversion test (*p* > 0.05), which may suggest that the red dawn/dusk transitions primarily influenced tonic, dispositional fear rather than reactive fear evoked by acute handling stress. Collectively, these findings extend prior work by Archer [2] and Remonato Franco et al. [3] demonstrating that lighting color and program design can modulate fear responses in broilers.

### 3.6. Illustrative Economic Analysis

To place these production differences into an economic context, an illustrative income-over-feed-cost (IOFC) partial-budget analysis was performed using representative, non-treatment-specific U.S. feed and live-bird prices (see Materials and Methods; Table 8). On a per-bird-marketed basis, DYN broilers returned an estimated $0.16/bird more than CON broilers in Experiment 1 (a 10.8% relative improvement), and RDW broilers returned an estimated $0.05/bird less than DYN broilers in Experiment 2 (a 3.5% relative reduction). When mortality was incorporated (IOFC per bird placed), the DYN advantage over CON narrowed to an estimated $0.09/bird placed (6.6%), because DYN broilers had lower livability than CON broilers (89.17 vs. 92.71%); the RDW reduction relative to DYN narrowed slightly to an estimated $0.04/bird placed (2.8%). These illustrative results suggest that, under representative market prices, the improved growth and feed efficiency of the DYN program would be expected to translate into a modest economic advantage over the conventional program, and that adding the red dawn/dusk transition (RDW) would be expected to slightly reduce feed-cost margin relative to DYN alone despite its skeletal and stress-related benefits. Because feed and live-bird prices vary substantially by region, time, and production contract, and because costs beyond feed (e.g., chick, labor, energy, housing, health) were not evaluated, these estimates should be interpreted only as an illustration of direction and relative magnitude, not as a validated economic recommendation; treatment-specific economic evaluation under commercial conditions and current local prices is needed before economic conclusions can be drawn.

## 4. Conclusions

1. An integrated dynamic LED lighting program (DYN) that combined a blue-emphasis spectrum, a declining light intensity, and a gradually shortening photoperiod (lengthening dark period) improved d-42 body weight, feed conversion ratio, and intestinal morphology, and markedly reduced physiological indicators of stress and fear in commercial Cobb 500 broilers compared to a conventional lighting program; because these lighting components changed simultaneously, this conclusion applies to the integrated program and should not be attributed to any single component (e.g., blue spectrum alone).
2. Adding a gradual red-light dawn/dusk transition to the DYN program (RDW) was associated with further increases in tibia breaking strength and reductions in corticosterone, heterophil-to-lymphocyte ratio, and asymmetry scores relative to the DYN program alone, without compromising growth performance; because the red dawn/dusk transition was itself confounded with the introduction of gradual light-to-dark transitions, these effects cannot be attributed to the red spectrum specifically.
3. The widespread adoption of programmable LED technology makes the implementation of broiler-targeted dynamic lighting programs increasingly accessible; the illustrative partial-budget analysis presented here (based on representative, not treatment-specific, feed and live-bird prices) suggests the DYN and RDW programs are unlikely to increase feed cost relative to a conventional program, but installation cost, energy consumption, and treatment-specific economic return were not evaluated and should be assessed under commercial-scale validation before economic feasibility can be concluded.
4. Future research should elucidate the physiological mechanisms underlying the skeletal and gastrointestinal effects observed, optimize transition period parameters, and validate these findings under commercial production conditions at scale.

## Figures and Tables

**Table 1:** Photoperiod, Intensity, and Color Temperature (K) of the Traditional Lighting Program (CON).

Day	Photoperiod	Lux	Kelvin
0	24L:0D	25	2700
1–5	23L:1D	25	2700
6–20	16L:8D	5	5000
21–27	17L:7D	5	5000
28–34	18L:6D	5	5000
35–38	19L:5D	5	5000
39	20L:4D	5	5000
40	21L:3D	5	5000
41	22L:2D	5	5000
42	23L:1D	5	5000

**Table 2:** Photoperiod and Intensity (lux) of the Dynamic Lighting Program (DYN).

Day	Photoperiod	Lux
0	24L:0D	75
1	23L:1D	75
2	23L:1D	75
3	23L:1D	75
4	22.67L:1.33D	75
5	22.33L:1.67D	75
6	22L:2D	75
7	21.67L:2.33D	70.4
8	21.33L:2.67D	65.7
9	21L:3D	61.1
10	20.67L:3.33D	56.4
11	20.33L:3.67D	51.8
12	20L:4D	47.2
13	19.67L:4.33D	42.5
14	19.33L:4.67D	37.9
15	19L:5D	33.2
16	18.67L:5.33D	28.6
17	18.67L:5.33D	23.9
18	18.33L:5.67D	19.3
19	18.33L:5.67D	14.7
20–42	18L:6D	10

**Table 3:** Ingredient and Calculated Nutrient Composition (%, as-fed basis unless otherwise stated) of the Starter, Grower, and Finisher Diets Fed in Experiments 1 and 2.

Item	Starter	Grower	Finisher
Ingredient (%, as-fed)			
Corn	52.69	57.48	61.87
Soybean meal (48% CP)	35.47	30.33	25.53
Distillers dried grains with solubles	5.00	5.00	5.00
Soybean oil	2.36	3.09	3.88
Monocalcium/dicalcium phosphate	1.62	1.44	1.25
Limestone	1.61	1.47	1.34
DL-Methionine	0.32	0.29	0.26
Vitamin premix ^1^	0.25	0.25	0.25
Salt	0.24	0.25	0.24
L-Lysine HCl	0.23	0.22	0.21
L-Threonine	0.13	0.11	0.08
Trace mineral premix ^1^	0.05	0.05	0.05
Sodium bicarbonate	0.03	0.03	0.04
Calculated nutrient composition			
AME (kcal/kg)	3000	3100	3200
Crude protein (%)	23.56	21.44	19.45
Crude fat (%)	4.96	5.80	6.68
Crude fiber (%)	2.46	2.39	2.33
Calcium (%)	0.96	0.87	0.78
Available phosphorus (%)	0.48	0.44	0.39
Total phosphorus (%)	0.74	0.68	0.62
Sodium (%)	0.16	0.16	0.16
Potassium (%)	0.88	0.79	0.72
Chloride (%)	0.23	0.23	0.23
Choline (mg/kg)	2265	2171	2083
Total lysine (%)	1.43	1.28	1.14
Total methionine + cysteine (%)	1.05	0.96	0.88
Digestible lysine (%)	1.28	1.15	1.02
Digestible methionine (%)	0.65	0.59	0.54
Digestible methionine + cysteine (%)	0.95	0.87	0.80
Digestible threonine (%)	0.86	0.77	0.68
Digestible tryptophan (%)	0.28	0.24	0.21
Digestible arginine (%)	1.38	1.23	1.09
Digestible isoleucine (%)	0.87	0.79	0.71
Digestible valine (%)	0.96	0.88	0.80

^1^ The trace mineral premix added at this rate yielded 149.6 mg manganese, 125.1 mg zinc, 16.5 mg iron, 1.7 mg copper, 1.05 mg iodine, 0.25 mg selenium, a minimum of 6.27 mg calcium, and a maximum of 8.69 mg calcium per kg of diet. The carrier was calcium carbonate, and the premix contained less than 1% mineral oil. Vitamin premix added at this rate yields 8818 IU vitamin A, 3086 IU vitamin D3, 37 IU vitamin E, 0.0132 mg B12, 4.676 mg riboflavin, 36.74 mg niacin, 16.17 mg d-pantothenic acid, 382.14 mg choline, 1.18 mg menadione, 1.4 mg folic acid, 5.74 mg pyridoxine, 2.35 mg thiamine, and 0.44 mg biotin per kg of diet. Diets were formulated by least-cost formulation (Concept5, CFC Tech Services, Pierz, MN, USA) to breeder specifications; AME = apparent metabolizable energy; digestible amino acids are standardized ileal digestible values. CP = crude protein.

**Table 4:** Production Parameters and Health Measurements of Broilers Reared Under Traditional (CON) or Dynamic (DYN) Lighting Programs: Experiment 1.

Parameter	Treatment	Mean ± SEM	*p*-Value
d-42 Body Weight (kg)	CON	2.85 ± 0.038	0.016
	DYN	2.99 ± 0.044	
Average Daily Gain (kg)	CON	0.066 ± 0.00091	0.016
	DYN	0.070 ± 0.0011	
Average Daily Feed Intake (g/bird/d)	CON	101.38 ± 1.62	0.07
	DYN	96.55 ± 2.09	
Feed Conversion Ratio	CON	1.53 ± 0.017	0.003
	DYN	1.45 ± 0.021	
Tibia Breaking Strength (g)	CON	31,452 ± 1758	>0.05
	DYN	30,083 ± 1693	
Bone Ash Content (%)	CON	53.98 ± 0.78	>0.05
	DYN	53.91 ± 0.34	
White Blood Cell Count (×10^3^/µL)	CON	23.21 ± 1.22	0.05
	DYN	19.68 ± 1.31	
Villus-to-Crypt Ratio	CON	1.053 ± 0.017	0.049
	DYN	1.369 ± 0.157	
Livability (%) ^1^	CON	92.71	
	DYN	89.17	
European Production Efficiency Factor (EPEF)	CON	411.2	
	DYN	437.8	

^1^ Livability (100 − mortality %) across the 42-d period; 35 and 52 dead or culled birds of 480 placed for CON and DYN, respectively (pen-level ANOVA *p* = 0.14; chi-square test on pooled counts *p* = 0.056; see Section 3.1).

**Table 5:** Fear and Stress Measurements of Broilers Reared Under Traditional (CON) or Dynamic (DYN) Lighting Programs: Experiment 1.

Parameter	Treatment	Mean ± SEM	*p*-Value
Corticosterone (pg/mL)	CON	2140.3 ± 340.1	0.01
	DYN	1129.8 ± 174.9	
Heterophil-to-Lymphocyte Ratio	CON	1.90 ± 0.34	0.006
	DYN	0.91 ± 0.08	
Composite Asymmetry Score (mm)	CON	1.76 ± 0.09	0.007
	DYN	1.43 ± 0.08	
TI Latency to Right (s)	CON	272.2 ± 20.3	0.011
	DYN	205.3 ± 16.3	
TI First Head Movement (s)	CON	98.8 ± 12.7	0.192
	DYN	120.6 ± 10.7	
TI Number of Attempts	CON	1.67 ± 0.07	0.006
	DYN	1.42 ± 0.06	
Inversion Wing Flaps	CON	17.5 ± 0.7	0.015
	DYN	20.4 ± 1.0	
Inversion Duration (s)	CON	3.60 ± 0.12	<0.001
	DYN	4.43 ± 0.19	
Inversion Intensity (flaps/s)	CON	4.94 ± 0.12	0.02
	DYN	4.56 ± 0.11	

**Table 6:** Production Parameters and Health Measurements of Broilers Reared Under Dynamic (DYN) or Dynamic + Red Dawn/Dusk (RDW) Lighting Programs: Experiment 2.

Parameter	Treatment	Mean ± SEM	*p*-Value
d 35 Body Weight (kg)	DYN	2.50 ± 0.05	0.941
	RDW	2.50 ± 0.03	
Average Daily Gain (g)	DYN	71.30 ± 1.39	0.941
	RDW	71.41 ± 0.77	
Average Daily Feed Intake (g/bird/d)	DYN	102.57 ± 0.95	0.018
	RDW	105.94 ± 0.99	
Feed Conversion Ratio	DYN	1.51 ± 0.02	0.123
	RDW	1.56 ± 0.02	
Tibia Breaking Strength (g)	DYN	30,650 ± 1539	<0.001
	RDW	38,951 ± 1006	
Bone Ash Content (%)	DYN	48.01 ± 0.34	0.529
	RDW	48.33 ± 0.36	
White Blood Cell Count (×10^3^/µL)	DYN	14.84 ± 1.03	0.13
	RDW	17.32 ± 1.15	
Villus-to-Crypt Ratio	DYN	4.16 ± 0.17	0.058
	RDW	3.75 ± 0.13	
Livability (%) ^1^	DYN	93.13	
	RDW	93.75	
European Production Efficiency Factor (EPEF)	DYN	440.5	
	RDW	429.3	

^1^ Livability (100 − mortality %) across the 35-d period; 33 and 30 dead or culled birds of 480 placed for DYN and RDW, respectively (pen-level ANOVA *p* = 0.77; chi-square test on pooled counts *p* = 0.70).

**Table 7:** Fear and Stress Measurements of Broilers Reared Under Dynamic (DYN) or Dynamic + Red Dawn/Dusk (RDW) Lighting Programs: Experiment 2.

Parameter	Treatment	Mean ± SEM	*p*-Value
Corticosterone (pg/mL)	DYN	25,062.6 ± 4319.7	0.016
	RDW	11,648.4 ± 3195.0	
Heterophil-to-Lymphocyte Ratio	DYN	0.81 ± 0.05	<0.001
	RDW	0.53 ± 0.04	
Composite Asymmetry Score (mm)	DYN	1.77 ± 0.09	0.018
	RDW	1.50 ± 0.08	
TI Latency to Right (s)	DYN	255.1 ± 19.6	0.031
	RDW	201.3 ± 15.2	
TI First Head Movement (s)	DYN	185.0 ± 18.4	0.017
	RDW	131.1 ± 13.0	
TI Number of Attempts	DYN	1.40 ± 0.05	0.027
	RDW	1.26 ± 0.05	
Inversion Wing Flaps	DYN	20.4 ± 0.9	0.94
	RDW	20.5 ± 0.8	
Inversion Duration (s)	DYN	3.76 ± 0.15	0.36
	RDW	3.95 ± 0.15	
Inversion Intensity (flaps/s)	DYN	5.50 ± 0.15	0.17
	RDW	5.25 ± 0.11	

**Table 8:** Illustrative Income-Over-Feed-Cost (IOFC) Partial-Budget Analysis Using Representative U.S. Feed and Live-Bird Prices ($0.38/kg feed; $1.10/kg live weight).

Exp.	Treatment	Final BW (kg)	FCR	Est. Feed Cost ($/Bird)	Est. Revenue ($/Bird)	Est. IOFC ($/Bird Marketed)	Livability (%)	Est. IOFC ($/Bird Placed)
1	CON	2.85	1.53	1.63	3.14	1.50	92.71	1.39
	DYN	2.99	1.45	1.62	3.29	1.66	89.17	1.48
2	DYN	2.50	1.51	1.41	2.75	1.34	93.13	1.25
	RDW	2.50	1.56	1.46	2.75	1.29	93.75	1.21

Exp. = Experiment; BW = body weight; FCR = feed conversion ratio; IOFC = income-over-feed-cost.

## Data Availability

The original contributions presented in this study are included in the article. Further inquiries can be directed to the corresponding author.

## Abbreviations

The following abbreviations are used in this manuscript:

CON	Conventional (traditional) lighting program
DYN	Dynamic (broiler-targeted) LED lighting program
RDW	Dynamic lighting program with red dawn/dusk transition
LED	Light-emitting diode
EPEF	European Production Efficiency Factor
IOFC	Income-over-feed-cost
TI	Tonic immobility
HL	Heterophil-to-lymphocyte ratio
CORT	Corticosterone
WBC	White blood cell count
ELISA	Enzyme-linked immunosorbent assay
EDTA	Ethylenediaminetetraacetic acid
ANOVA	Analysis of variance
GLM	General linear model
SEM	Standard error of the mean
IACUC	Institutional Animal Care and Use Committee
AUP	Animal Use Protocol
